# Editorial: Advanced learning control in physical interaction tasks

**DOI:** 10.3389/frobt.2023.1166759

**Published:** 2023-02-24

**Authors:** Chao Zeng, Jing Guo, Qiang Li, Chenguang Yang

**Affiliations:** ^1^ Department of Informatics, TAMS group, Universität Hamburg, Hamburg, Germany; ^2^ School of Automation, Guangdong University of Technology, Guangzhou, China; ^3^ Bristol Robotics Laboratory, University of the West of England, Bristol, United Kingdom

**Keywords:** robotics, learning control, physical interaction, robot learning, dexterous manipulation

Robotics are increasingly and urgently expected to acquire human-like dexterous manipulation skills in physical interaction environments. Due to this, the loop between the high-level action policy and the low-level motion execution needs to be closed by developing advanced data-driven or model-based learning and control approaches. Although recent studies have been shown to demonstrate promising results and advances in closed-loop learning control algorithms, several key Research Topic remain quite a changeling. Typically, three problems in this field of research are yet to be solved: 1) how to integrate learning and control models seamlessly for more dexterous manipulation and interaction performances; 2) how to compute learning and control policies from multi-modal/cross-modal data; and 3) how to make use of advances in both data-driven and model-based models for compliant and flexible interactions. We publish this Research Topic to bring together the newest theoretical findings and experimental results in advanced learning control applied to robot-environment physical interaction systems. Five manuscripts out of all submissions to this special Research Topic are accepted after a standard review process. Below we give a brief review of the published articles.

Qin et al. summarize the state-of-the-art research on robotic tool usage, which is of great importance in physical interaction tasks. In this survey, they first give the definition of robot tool usage that is necessary to understand the uniqueness of tool use. Then, they present a taxonomy of robot tool usage inspired by animal tool use. Subsequently, the skills required for robot tool usage are summarized. Next, they review the robot tool use literature in detail based on the definition and taxonomy of robot tool usage from three aspects: 1) non-causal tool use; 2) causal tool use—single-manipulation tool use; and 3) causal tool use—multiple-manipulation tool use. They finally discuss the current applications of robot tool usage as well as the open challenges of future research on this Research Topic. Saha et al. propose the MoViLan, a modular vision language navigation and manipulation framework, to deal with long horizon compositional tasks in indoor environments. Unlike most previous approaches, this framework handles the combined navigation and object interaction problem without the requirement that the vision and language training data must be strictly aligned, and there it allows for a more convenient training process with separate vision and language datasets. Within this framework, they also propose a geometry-aware mapping technique for cluttered indoor environments and a language-understanding model for robot navigation and manipulation by following household instructions. Zito and Ferrante propose a one-shot learning approach for autonomous aerial physical manipulation. Their approach allows one to learn the probability density of contacts over the surface of an object based on a single demonstration and further enhances the formulation for learning aerial transportation tasks without the need of handcrafting task-dependent features. Furthermore, their approach only depends on the geometrical properties of the payloads, which can be computed from a point cloud that is robust to partial views. Gabler and Wollherr propose a novel approach for robot learning compliant skills in force-sensitive contact-rich tasks, based on meta-learning for graphical skill-formalism. They propose to use a hybrid force–velocity controller to design a graphical skill-formalism, which incorporates available task knowledge and allows for online episodic reinforcement learning (RL). They further extend this skill-formalism by estimating the success probability of the task that will be learned by the means of factor graphs. They also propose suitable constraint Gaussian Process (GP) models and acquisition functions to optimize the gain of the controller, while considering the task success probability. Rizzardo et al. attempt to address the sim-to-real gap by developing a sim-to-real approach that trains a Soft-Actor Critic (SAC) agent with a decoupled feature extractor and a latent-space dynamics model. The former allows for the sim-to-real transfer of the feature extractor and control policy in an independent manner, and the latter is used as a constraint on the latent representation when fine-tuning the feature extractor in reality. The advantage of their approach is that fine-tuning the control policy is not needed when transferring the learned agent from simulation to reality, thus one can only focus on the adaptation of the feature extractor using real-world data, in order to improve the efficiency of sim-to-real transfer.

Finally, we expect that this Research Topic can provide inspiration for the research on robot learning and control for physical interaction task scenarios.

